# Predictive Factors for Successful Cervical Ripening among Women with Gestational Diabetes Mellitus at Term: A Prospective Study

**DOI:** 10.3390/jcm13010139

**Published:** 2023-12-26

**Authors:** Guillaume Ducarme, Lucie Planche, Mounia Lbakhar

**Affiliations:** 1 Department of Obstetrics and Gynecology, Centre Hospitalier Departemental, 85000 La Roche sur Yon, France; mounia.lbakhar@gmail.com; 2 Clinical Research Center, Centre Hospitalier Departemental, 85000 La Roche sur Yon, France; lucie.planche@ght85.fr

**Keywords:** gestational diabetes mellitus, Bishop score, cervical ripening, induction of labor, vaginal delivery, predictive factors

## Abstract

The purpose of this prospective cohort study is to identify the predictive factors for vaginal delivery among women (n = 146) who underwent cervical ripening using a dinoprostone insert (PG) alone (13.7%), cervical ripening balloon (CRB) alone (52.7%), oral misoprostol (M) alone (4.1%), or repeated methods (R, 29.5%) for gestational diabetes mellitus (GDM) at term, and to analyze maternal and neonatal morbidity outcomes according to the method for cervical ripening. After cervical ripening, vaginal delivery occurred in 84.2% (n = 123) and was similar among groups (90.0% after PG, 83.1% after CRB, 83.3% after M, and 83.7% after R; *p* = 0.89). After a multivariable logistic regression analysis adjusted for potential confounders, the internal cervical os being open before cervical ripening was a predictor of vaginal delivery (adjusted odds ratio (OR) of 4.38, 95% confidence index (CI) of 1.62–13.3, *p* = 0.03), and previous cesarean delivery was a predictor of cesarean delivery (aOR of 7.67, 95% CI of 2.49–24.00, *p* < 0.01). Birthweight was also significantly associated with cesarean delivery (aOR of 1.15, 95% CI of 1.03–1.31, *p* = 0.02). The rates of maternal and neonatal morbidity outcomes were 10.9% (n = 16) and 19.9% (n = 29), respectively, and did not differ according to the mode of delivery and to the method used for cervical ripening. Identifying these specific high-risk women (previous cesarean delivery and internal cervical os being closed before cervical ripening) for cesarean delivery among women who underwent cervical ripening for GDM at term is important and practical for all physicians to make a decision in partnership with women.

## 1. Introduction

Gestational diabetes mellitus (GDM) is defined as any degree of glucose intolerance with an onset or first recognition during pregnancy [1,2]. In women with GDM, there is evidence of major changes in the mother, placental, and fetal metabolism, as well as implications for fetal growth and development. Impaired pancreatic insulin secretion, dyslipidemia, and defects in the insulin signaling pathway in the skeletal muscle and adipose tissue that relate to the altered glucose–insulin handling and a higher prevalence of increased fetal adiposity and fetal macrosomia were observed in women with GDM [3,4,5,6].

Improved glycemic control among women with GDM allows to reduce maternal mortality and morbidity due to labor complications (severe perineal tears, postpartum hemorrhage, and cesarean delivery (CS)) and also to reduce neonatal mortality and morbidity (macrosomia, shoulder dystocia, brachial plexus injury, and neonatal asphyxia) [7,8,9,10,11,12]. The current guidelines for the management of GDM consider that, in women who present with a strict glycemic control with diet alone or with antenatal insulin therapy (AIT), there is no argument that justifies a different management from a normal pregnancy and allows awaiting spontaneous labor until 41 weeks. Women who are not controlled with AIT or who present with suspected fetal macrosomia at 37 weeks (defined as an ultrasonographic-estimated fetal weight (EFW) > 97th centile) are advised to undergo induction of labor (IOL) at 39 weeks to reduce potential stillbirths and neonatal morbidity (macrosomia, shoulder dystocia, brachial plexus injury, and neonatal asphyxia) [1,2]. GDM has become the most important cause for IOL all around the world. A recent study based on nationwide data from the Icelandic Medical Birth Register on 85,620 singleton births from 1997 to 2018 showed that IOL was increasingly indicated from 1997–2001 to 2014–2018 mostly because of GDM (2.4% to 16.5%) [13].

Many data comparing different methods for IOL are available and showed that mechanical induction with a balloon catheter is probably as effective as vaginal prostaglandins with a more favorable safety profile; however, it may be slightly less effective than oral misoprostol or low-dose vaginal misoprostol, but with a better safety profile [14,15,16,17,18,19,20]. Nevertheless, there are insufficient comparative data to determine which of the methods is the most effective and safe for women with GDM at term [21]. Effectively, some physiopathology findings in women with GDM must be taken into consideration, such as the impairment of uterine contractility with significant decreased uterine contraction amplitude and duration due to reduced calcium channel expression and signaling, which may lead to a poorer myometrial activity during labor and an increased risk of CS [7]. Moreover, variation in parity, gestational age at IOL, as well as other demographic features of women with GDM (higher body mass index (BMI), greater maternal age, more nulliparity, more frequent polyhydramnios, and higher birthweight) may undoubtedly affect the clinical results. Some studies showed that the duration of labor, the mode of delivery, and maternal and neonatal outcomes seemed to not be impacted by GDM [22,23,24], while other published studies reported a significantly longer time in active labor [25] or an increased risk of CS after IOL among women with GDM, compared to the general population [26,27,28,29]. In order to try to understand the nuances in safety and efficacy outcomes, a prospective observational study may assess which method is associated more with vaginal delivery among these specific women, to look for the optimal protocol of cervical ripening among women with GDM.

The aim of this prospective observational study is to identify the predictive factors for vaginal delivery among women who required cervical ripening for GDM at term and to analyze maternal and neonatal morbidity outcomes according to the mode of delivery and to the device used.

## 2. Materials and Methods

### 2.1. Study Design

This was a planned secondary analysis of a prospective cohort study that included all consecutive women who underwent cervical ripening using a dinoprostone insert (PG), cervical ripening balloon (CRB), oral misoprostol (M), or repeated methods (R) from 9 January 2020 to 30 June 2021 in a tertiary care university hospital with more than 2600 annual deliveries [30].

### 2.2. Participants

For the study, we included women with live singleton fetuses at term (>37 weeks of gestation) in vertex presentation and intact membranes who underwent cervical ripening using PG, CRB, M, or R for GDM that was not controlled by AIT or that was associated with suspected fetal macrosomia at 37 weeks. The exclusion criteria were women aged < 18 years, with previous diabetes, a first-trimester fasting plasma glucose (FPG) ≥ 126 mg/dL, multiple gestation, women without ultrasound gestational age dating before 24 weeks (according to crown-to-rump length in the first trimester or fetal biometry in the second trimester), placenta closer than 2 cm from the internal os, undiagnosed vaginal bleeding, active or purulent infection of the lower genital tract, lethal congenital anomaly, allergy to latex, and non-reassuring pre-ripening cardiotocograph (CTG).

### 2.3. Ethical Approval

The present study was conducted in accordance with the principles of the Declaration of Helsinki and French approved guidelines. Information on the study was provided to the eligible women by obstetricians and midwives in the labor ward immediately before cervical ripening. All participants received written information about the study using institutional review board-approved documents. A written consent is not required for prospective observational studies according to the French law, but each eligible woman was provided with the opportunity to decline being included in the prospective cohort study. After providing written information and oral consent, an anonymous number was allocated to each included woman. The study protocol was approved by a Research Ethics Committee (*Groupe Nantais d’Ethique dans le Domaine de la Santé* (GNED)) on 8 January 2020 before the beginning of the study (ethical code number: 2020-01-08).

### 2.4. Procedures

Details of the procedures used to manage cervical ripening and labor, maternal characteristics, intrapartum variables, and clinical outcomes identified in the immediate postpartum period were prospectively collected using a maintained database of the women who were included in the study [30].

As described in detail previously [30] and according to the French guidelines [1], pregnant women who belong to a risk group (e.g., age ≥ 35 years, BMI ≥ 25 kg/m^2^, familial history of diabetes, and previous GDM or macrosomia) undergo a FPG during the first trimester of the pregnancy. GDM is diagnosed if FPG meets or exceeds 92 mg/dL (5.1 mmol/L) [1,2]. Women who present with the risk factors of GDM and a normal first-trimester FPG undergo a 75 g oral glucose tolerance test between 24 and 28 weeks. GDM is diagnosed if one or more plasma glucose levels meet or exceed the recommended thresholds [1,2].

As described in detail previously [31,32], after being diagnosed with GDM, all women were admitted in an ambulatory procedure and managed with education, lifestyle intervention, and medical nutrition therapy to maintain strict glycemic control using self-monitoring of blood glucose values (preprandial goal < 95 mg/dL and postprandial goal < 120 mg/dL at 2 h [1,2]). AIT was introduced after 1 or 2 weeks of failed achievement of glycemic control. In our center, as described previously [31,32], all women with GDM underwent a fetal ultrasound assessment at 22, 28, 32, and 36 weeks. Women who were not controlled with AIT or who presented with suspected fetal macrosomia at 37 weeks (defined as an ultrasonographic EFW > 97th centile [33]) were advised to undergo IOL at 39 weeks to reduce potential stillbirths and neonatal morbidity (macrosomia, shoulder dystocia, brachial plexus injury, and neonatal asphyxia) [1,2]. The others were allowed to await spontaneous labor until 41 weeks [1,2]. No variation in practice among the women was observed throughout the period of study, as the same team cared them.

In women with an unfavorable cervix (Bishop score < 6), cervical ripening by mechanical (CRB) or pharmacologic (PG or M) methods prior to the onset of spontaneous labor is advised to improve the vaginal delivery rate. In our center, the choice of the cervical ripening method was left to the free discretion of the obstetrician in accordance with the institutional local guidelines [30]. All women were admitted to the labor ward and kept there for at least 30 min of a normal CTG before (and after) undergoing the cervical ripening method that was used, as per the manufacturer’s instructions (Cervical Ripening Balloon^®^; Cook OB/GYN, Spencer, IN, USA; Propess^®^; Ferring, Saint-Prex, Switzerland, and Angusta^®^; Norgine B.V., Amsterdam, The Netherlands). CRB and PG were used for a maximum period of 24 h, and were removed before 24 h in the case of spontaneous labor, expulsion, non-reassuring CTG result, spontaneous rupture of membranes, or unexplained vaginal bleeding. Oral misoprostol (M) was continued for more than 24 h. If labor did not ensue or the Bishop score was still unfavorable (score < 6) after 24 h, there was no consensus, and repeating cervical ripening was proposed (R group). Once the Bishop score was ≥6, further management with amniotomy and oxytocin induction was recommended in our team.

### 2.5. Data Collection

As described in detail previously [30], maternal characteristics (age, pre-pregnancy weight and BMI, and women’s medical and obstetric history (i.e., previous CS)) were collected throughout the study period. Data about pregnancy and labor characteristics were also collected: antenatal suspicion of macrosomia (defined as ultrasonographic EFW > 97th centile at 37 weeks), cervical ripening method, Bishop score and each Bishop score criterion (dilatation, effacement, fetal station, consistency, and position) before and after ripening, uterine tachysystole, artificial rupture of membranes before starting oxytocin, mode of labor (oxytocin induction, quantity of oxytocin used, and amniotomy), gestational age at delivery, mode of delivery (spontaneous or operative vaginal delivery and CS), the indication of CS, the time of CS (before labor (failure of IOL) or during labor (during the latent or active phase)), birth weight, and immediate maternal and neonatal outcomes. The maternal outcomes included the severity of perineal tears, postpartum hemorrhage (PPH, defined as bleeding 500 mL or greater, with routinely assessed blood loss with a collector bag placed just after birth), severe PPH (defined as bleeding 1000 mL or greater), the need for additional uterotonic agent (sulprostone) and second-line therapies (Bakri balloon, uterine compression sutures, uterine artery embolization, and peripartum hysterectomy) for the management of massive persistent PPH after the failure of uterine massage and uterotonic agents to stop bleeding, chorioamnionitis (defined as maternal hyperthermia greater than 38.5 °C, maternal tachycardia greater than 100 bpm, fetal tachycardia greater than 160 bpm, and meconium-stained amniotic fluid), infections (defined as at least one of the following: endometritis, episiotomy infection, or wound infection requiring surgery), blood transfusion, thromboembolic event, intensive care unit (ICU) admission, and maternal death.

Neonatal immediate outcomes included umbilical arterial blood gases at birth, 5 min Apgar score, shoulder dystocia (defined as the application of additional obstetric maneuvers following the failure of gentle downward traction on the fetal head to enable the delivery of the fetal shoulders [34,35]), neonatal trauma (defined as the existence of at least one of the following: the fracture of the clavicle or a long bone, brachial plexus injury, or cephalhematoma), respiratory distress syndrome, neonatal jaundice (that needed phototherapy after birth), neonatal hypoglycemia (defined as blood glucose < 40 mg/dL in the first 24 h post-delivery or blood glucose < 50 mg/dL from the second day of life), intraventricular hemorrhage, the need for resuscitation or intubation, any transfer to the neonatal intensive care unit (NICU) for the close monitoring of the neonate, sepsis (defined as confirmed clinical infection with positive bacteriological tests), seizures, and neonatal death.

### 2.6. Endpoints

The primary endpoint was vaginal delivery after cervical ripening. The secondary outcomes were composite maternal and neonatal morbidity. Maternal morbidity was defined by at least one of the following criteria: chorioamnionitis, third- or fourth-degree perineal tears, PPH, need for sulprostone, second-line therapies, blood transfusion, ICU admission, and maternal death. Neonatal morbidity was a composite variable, defined by at least one of the following criteria: shoulder dystocia, 5 min Apgar score less than 7, pH less than 7.10, respiratory distress syndrome, neonatal hypoglycemia, neonatal trauma, neonatal hyperbilirubinemia, sepsis, intraventricular hemorrhage greater than grade 2, seizures, need for resuscitation or intubation, NICU admission, and neonatal death.

### 2.7. Statistical Analysis

Continuous data were described by their means ± standard deviations and compared by *t*-tests (or Mann–Whitney tests when appropriate), and categorical data were described by percentages and compared using chi-squared tests (or Fisher’s exact tests when appropriate). The predictive factors for vaginal delivery after cervical ripening among women with GDM at term were analyzed using appropriate tests. We also compared the maternal and neonatal outcomes according to the mode of delivery, and specifically studied the association (assessed by multivariate logistic regression analyses) among the mode of delivery, maternal and neonatal morbidity outcomes, and the method used for cervical ripening (PG, CRB, M, or R). The multivariate logistic regression allowed us to analyze (together) the effect of other risk factors and potential confounders (maternal age, parity, BMI before pregnancy, gestational age at delivery, mode of delivery, and birth weight). No formal sample size was calculated as we collected data for all cases over a specified time period. We used the R software (version 4.2.0 Patched) for all the analyses. A *p*-value < 0.05 was considered statistically significant.

## 3. Results

During the study period, 3920 births took place in our tertiary public hospital, and 718 women (18.3%) with a live singleton fetus at term in the vertex presentation required cervical ripening for different indications (maternal, fetal, or both) in our hospital. Among these women, 146 (20.3%) underwent cervical ripening at term due to GDM that was not controlled with AIT or due to suspected fetal macrosomia at 37 weeks. Among the 146 women included, 39.7% (n = 58) were nulliparous, and 61.6% (n = 90) required AIT for glycemic control during pregnancy. The maternal characteristics according to the mode of delivery are shown in Table 1.

The rates of obesity, nulliparity, previous GDM, GDM with AIT, and antenatal suspicion of macrosomia were similar among groups. The rate of women with previous CS was significantly higher among women who had CS compared to women who delivered vaginally (34.8% vs. 6.5%, *p* < 0.001). The mean gestational age at birth was 39.1 ± 0.6 weeks and was also similar among groups (*p* = 0.22) (Table 1). Concerning cervical ripening, there were 39.7% (n = 58) women with a Bishop score < 3 before cervical ripening. Among these included women, a dinoprostone insert alone (PG) was used by 13.7% (n = 20), a cervical ripening balloon alone (CRB) by 52.7% (n = 77), oral misoprostol alone (M) by 4.1% (n = 6), and 29.5% of women underwent repeated methods (R) (n = 43) for cervical ripening. These rates were similar according to the mode of delivery (Table 1). Among Bishop score criteria at enrollment, only one criterion, ‘dilatation’ (internal cervical os open), differed among the groups (65.0% in the vaginal delivery group vs. 26.1% in the CS group, *p* < 0.001). No uterine tachysystole was observed in the study. Twenty-four hours after cervical ripening, 45.2% (n = 66) women were in labor, 50.7% (n = 74) had IOL with amniotomy+/-oxytocin infusion due to a favorable Bishop score, and 4.1% (n = 6) required CS for IOL failure. A higher rate of labor after cervical ripening was reported among women who delivered vaginally (51.2% vs. 13.0%, *p* < 0.01). Vaginal delivery occurred in 84.2% (n = 123) and was similar regardless of the method of cervical ripening used (90.0% (18/20) after PG, 83.1% (64/77) after CRB, 83.3% (5/6) after M, and 83.7% (36/43) after R; *p* = 0.89) (Table 1).

Maternal adverse events were rare and similar among groups (Table 1). No blood transfusion, thromboembolic event, intensive care unit admission, or maternal death were observed in the study. Maternal morbidity among women who gave birth to a live singleton fetus after cervical ripening for GDM at term was 10.9% (n = 16) and was similar according to the mode of delivery used (10.6% after vaginal delivery vs. 13.0% after CS, *p* = 0.69) (Table 1).

After multivariable logistic regression analysis adjusted for parity, consistency and dilatation of the cervix before ripening, gestational age at birth, and birth weight, dilatation at enrollment (internal cervical os open) was a predictive factor of vaginal delivery (adjusted odds ratio (OR) of 4.38, 95% confidence index (CI) of 1.62–13.3, *p* = 0.03). Contrarily, previous CS was a predictive factor of CS (aOR of 7.67, 95% CI of 2.49–24.00, *p* < 0.01) after cervical ripening (Table 2).

We also observed, after multivariable logistic regression analysis adjusted for potential confounders (parity and gestational age at delivery), that birthweight was significantly associated with CS (aOR of 1.15, 95% CI of 1.03–1.31, *p* = 0.02) (Table 2).

The neonatal outcomes according to the mode of delivery are shown in Table 3. Birthweight was significantly higher after cesarean delivery compared to vaginal delivery (3649 ± 521 g vs. 3421 ± 403 g, *p* = 0.02). No 5 min Apgar score less than 7, seizures, intraventricular hemorrhage greater than grade 2, neonatal trauma, or neonatal death were observed in the study. Neonatal morbidity was 19.9% (n = 29) and did not differ according to the mode of delivery (19.5% after vaginal delivery vs. 21.7% after CS, *p* = 0.60) (Table 3).

The univariate and multivariate analyses of maternal and neonatal morbidity outcomes after cervical ripening among women with GDM showed that maternal and neonatal morbidity outcomes were not significantly different according to the method for cervical ripening (Table 4).

## 4. Discussion

In our study, dilatation (internal cervical os open) before cervical ripening was a predictive factor of vaginal delivery among women who underwent cervical ripening methods for GDM at term. We also observed that previous CS and birthweight were significantly associated with CS among these women. No factor was associated with either maternal or neonatal morbidity.

Our study did not show that the Bishop score before cervical ripening was as a predictive factor for vaginal delivery, while dilatation (internal cervical os open) before cervical ripening was an important predictive factor of vaginal delivery. These data are in agreement with those in the literature [36,37]. Lyndrup et al. [36] demonstrated that the Bishop score may be replaced when cervical ripening is required, without taking into account the position and consistency of the cervix, but that more importance should be placed on cervical dilatation. Ivars et al. [37] also found that dilatation (internal cervical os open) before cervical ripening, parity, and fetal position are the main predictive factors, but not the Bishop score.

Although data comparing different methods for IOL are available [14,15,16,17,18,19,20], there are insufficient comparative data to determine which of the methods is the most effective and the safety profile among women with GDM at term. Moreover, in the published studies, the greater maternal age, higher BMI, higher nulliparity, and higher birthweight in the included women with GDM, compared to the general population, may undoubtedly affect the clinical results. Three retrospective studies with many bias investigated whether GDM itself had an effect on length of labor, mode of delivery, and maternal and neonatal outcomes among women with GDM compared with women without GDM in spontaneous labor at term [22], or by comparison with women without GDM and cervical ripening at term for another maternal and/or fetal indication [23], or by comparison with women with controlled GDM with diet alone [24]. A post hoc analysis using pooled data of two large multicenter randomized-controlled trials investigating the use of a dinoprostone vaginal insert for cervical ripening, which analyzed the duration of labor induction between diabetic and nondiabetic women that received a dinoprostone vaginal insert, was also published in 2021 [38]. These studies clearly demonstrated that, even if women with GDM were different than other women (higher BMI, greater maternal age, more nulliparity, and higher birthweight), the duration of labor, the mode of delivery, and the maternal and neonatal outcomes were not impacted by GDM [31,32,33], except in one study that included women with GDM as well as pre-pregnancy type 1 and 2 diabetes that limited the interpretations of the results [38]. The largest retrospective study in China (226 women with GDM and cervical ripening, and 1329 women without GDM and cervical ripening at term for another maternal and/or fetal indication) conducted a multivariate analysis that showed that the delivery rates of women with GDM delivered within 12, 24, 36, or 48 h and those delivered vaginally within 12 or 36 h were significantly lower than those of the controls, which clearly suggests the existence of other independent predictive factors beyond those studied [23]. A recent large population-based cohort study including 247,524 primiparous women who gave birth to a singleton fetus with cephalic presentation ≥ 34 weeks in Sweden showed that women with GDM underwent a significantly longer time in active labor with IOL and an increased risk of CS compared to women without GDM [25]. Nevertheless, few studies have assessed which method was associated more with vaginal delivery and less with maternal–fetal morbidity among women with GDM in order to look for the optimal protocol of cervical ripening for these specific women. Unfortunately, as reported in the data in the literature [18,20], we did not find any cervical ripening method that was more effective than the ones investigated in our study to increase the rate of vaginal delivery nor more likely to decrease perinatal morbidity.

Our study has several strengths. First, the study was a planned secondary analysis of data collected for a prospective observational study in a single site. Second, the strengths of this study lie in the homogeneous nature of the population studied (only women with live singleton fetuses at term in the vertex presentation and intact membranes who underwent cervical ripening for GDM that were not controlled by AIT or with an ultrasonographic EFW > 97th centile at 37 weeks were included), which increases its external validity and allows for a better extrapolation of the results. Third, all included women with GDM were cared for by the same obstetric and endocrinology team and were followed using standardized protocols including the need for treatment or obstetric intervention, and thus especially avoided significant differences in the outcomes due to variations in clinical practice. Fourth, another strength of this study is the use of multiple aspects for evaluating neonatal complications and a statistical analysis that accounted for multiple confounding factors.

Our results must be interpreted in light of certain limitations. First, our study reflects the experience of one tertiary hospital and the results may not be generalizable to all maternity wards using other practices for women with GDM (center effect bias). Second, no sample size to demonstrate the feasibility of the study was calculated before the study. The small sample size may mask the occurrence of rare but serious maternal–fetal events, shoulder dystocia, serious perineal tears, and hospitalization in an intensive care or in NICU, and therefore the small sample size of this study could have been too small to reveal a significant difference among the different ripening methods. Third, the present study did not specifically study the women’s experience and satisfaction of cervical ripening according to the different methods; thus, additional studies are necessary. Finally, the results might have been influenced by hidden confounders that were unfortunately not recorded, such as samples of women induced for glycemic imbalance and those induced for fetal impact.

## 5. Conclusions

The limitations notwithstanding, our study supports the continued use of the analysis of each Bishop score criteria before cervical ripening to predict vaginal delivery among women with GDM at term. Specifically, dilatation before cervical ripening (=internal cervical os open) was a predictive factor of vaginal delivery after cervical ripening among these women. Identifying specific high-risk women (previous CS and internal cervical os closed before cervical ripening) for CS among women who underwent cervical ripening for GDM at term is important and practical for all physicians to make a decision in partnership with women (i.e., methods for cervical ripening).

## Figures and Tables

**Table 1:** Maternal and labor characteristics, and maternal outcome according to the mode of delivery.

	Vaginal Delivery, n = 123 (84.2%)	Cesarean Delivery, n = 23 (15.8%)	*p*-Value
Maternal characteristics			
Age, years	31.8 ± 5.4	32.1 ± 5.9	0.80
Pre-pregnancy BMI, kg/m^2^	28.4 ± 6.6	30.9 ± 6.2	0.07
Obesity (BMI ≥ 30 kg/m^2^)	46 (37.4)	11 (47.8)	0.35
Tobacco use	17 (13.8)	1 (4.3)	0.30
Chronic hypertension	3 (2.4)	2 (8.7)	0.20
Nulliparity	45 (36.6)	13 (56.5)	0.07
Previous CS	8 (6.5)	8 (34.8)	<0.001
Previous GDM	34 (27.6)	4 (17.4)	0.30
Pregnancy characteristics			
ART	7 (5.7)	3 (13.0)	0.20
GDM with AIT	80 (65.0)	10 (43.5)	0.05
Gestational weight gain, kg	10 ± 6	12 ± 7	0.11
Pregnancy-associated hypertensive disorders	2 (1.6)	0	0.94
SGA	4 (3.3)	1 (4.3)	0.60
Antenatal suspicion of macrosomia	42 (34.1)	10 (43.5)	0.48
Cervical ripening characteristics			
Gestational age, weeks	39.1 ± 0.7	39.1 ± 0.6	0.89
Bishop score < 3	46 (37.4)	12 (52.2)	0.19
Bishop score criteria at enrollment			
Dilatation (internal cervical os open)	80 (65.0)	6 (26.1)	<0.001
Effacement (>30%)	65 (52.8)	15 (65.2)	0.27
Fetal station (-2 and lower)	77 (62.6)	15 (65.2)	0.81
Consistency (medium or soft)	73 (59.3)	12 (52.2)	0.52
Position (middle or anterior)	36 (29.3)	5 (21.7)	0.45
Cervical ripening			0.89
Dinoprostone insert alone	18 (14.6)	2 (8.7)	
Cervical ripening balloon alone	64 (52.0)	13 (56.5)	
Oral misoprostol alone	5 (4.1)	1 (4.4)	
Repeated methods	36 (29.3)	7 (30.4)	
Labor characteristics			
Mode of labor			<0.001
Labor after cervical ripening	63 (51.2)	3 (13.0)	
Artificial rupture of membranes before starting oxytocin	60 (48.8)	14 (60.9)	
CS for cervical ripening failure	0	6 (26.1)	
Epidural analgesia	109 (88.6)	18 (78.3)	0.21
Gestational age at birth, weeks	39.3 ± 1.0	39.4 ± 1.2	0.22
Maternal outcome			
PPH	10 (8.1)	3 (13.0)	0.41
Severe PPH	3 (2.4)	0	0.92
Episiotomy	21 (17.1)	4 (17.4)	0.94
Third- or fourth-degree perineal	2 (1.6)	0	0.94
Need for additional uterotonic agent (sulprostone)	1 (0.8)	1 (4.3)	0.32
Second-line therapies	3 (2.4)	0	0.92
Chorioamnionitis	2 (1.6)	0	0.94
Infections	0	1 (4.3)	0.95
Maternal morbidity	13 (10.6)	3 (13.0)	0.69

Values are presented as mean ± SD or number (percentage) unless otherwise indicated. BMI, body mass index; GDM, gestational diabetes mellitus; ART, assisted reproductive technology; AIT, antenatal insulin treatment; SGA, small for gestational age; CS, cesarean delivery; PPH, postpartum hemorrhage. Continuous data were compared using *t*-tests (or Mann–Whitney tests when appropriate), and categorical data were compared using chi-squared tests (or Fisher’s exact tests when appropriate). A *p*-value < 0.05 was considered statistically significant.

**Table 2:** Multivariate analysis of vaginal delivery after cervical ripening among women with GDM.

Variable	Vaginal Delivery (n = 123/146, 84.2%)	
	Adjusted OR (95% CI)	*p*-Value
Nulliparity	0.37 (0.12–1.06)	0.06
Previous CS	0.13 (0.04–0.40)	<0.001
Gestational age at delivery (for each week)	1.32 (0.59–2.93)	0.49
Dilatation at enrollment (internal cervical os open)	4.38 (1.62–13.3)	0.003
Birth weight (for each 100 g)	0.87 (0.76–0.97)	0.02

OR, odds ratio; CI, confidence interval; CS, cesarean delivery. Adjusted for parity, consistency and dilatation of the cervix before ripening, gestational age at delivery, and birth weight.

**Table 3:** Neonatal outcomes among women with GDM according to the mode of delivery.

	Vaginal Delivery, n = 123 (84.2%)	Cesarean Delivery, n = 23 (15.8%)	*p*-Value
Birth weight, g	3421 ± 403	3649 ± 521	0.02
pH less than 7.10	3 (2.4)	2 (8.7)	0.24
Need for resuscitation or intubation	1 (0.8)	1 (4.3)	0.32
Shoulder dystocia	4 (3.3)	0	0.96
Respiratory distress syndrome	8 (6.5)	1 (4.4)	0.91
Neonatal jaundice	4 (3.3)	2 (8.7)	0.23
Neonatal hypoglycemia	4 (3.3)	0	0.96
Sepsis	1 (0.8)	0	0.97
NICU admission	5 (4.1)	1 (4.4)	0.99
Neonatal morbidity	24 (19.5)	5 (21.7)	0.60

Values are presented as mean ± SD or number (percentage) unless otherwise indicated. NICU, neonatal intensive care unit. Continuous data were compared using *t*-tests (or Mann–Whitney tests when appropriate), and categorical data were compared using chi-squared tests (or Fisher’s exact tests when appropriate). A *p*-value < 0.05 was considered statistically significant.

**Table 4:** Univariate and multivariate analyses of maternal and neonatal morbidity outcomes after cervical ripening.

Variable	Maternal Morbidity					Neonatal Morbidity				
	No (n = 130)	Yes (n = 16)	*p*-Value	Adjusted OR (95% CI)	*p*-Value	No (n = 117)	Yes (n = 29)	*p*-Value	Adjusted OR (95% CI)	*p*-Value
Age, years	32.0 ± 5.6	30.7 ± 4.6	0.32	0.99 (0.89–1.10)	0.85	31.6 ± 5.5	33.1 ± 5.0	0.19	1.09 (0.99–1.20)	0.08
Pre-pregnancy BMI, kg/m^2^	28.9 ± 6.6	27.5 ± 6.2	0.41			30.9 ± 6.6	28.2 ± 6.6	0.12		
Obesity (BMI ≥ 30 kg/m^2^)	52 (40.0)	5 (31.2)	0.49			42 (35.9)	12 (41.3)	0.26		
Nulliparity	48 (36.9)	10 (62.5)	0.05	2.73 (0.88–9.24)	0.08	43 (36.7)	14 (48.3)	0.08	2.55 (0.92–7.53)	0.07
Previous CS	13 (10.0)	3 (18.7)	0.33			12 (10.2)	3 (10.3)	0.80		
Gestational weight gain, kg	10 ± 7	12 ± 6	0.29			11 ± 7	9 ± 5	0.18		
Bishop score at enrollment < 3	50 (38.5)	8 (50.0)	0.38			50 (42.7)	8 (27.6)	0.22		
Gestational age at delivery, weeks	39.1 ± 0.6	39.2 ± 0.7	0.77			39.1 ± 0.6	39.1 ± 0.8	0.60		
Method for cervical ripening			0.60					0.79		
Cervical ripening balloon alone	67 (51.5)	10 (62.5)				61 (52.1)	16 (55.2)			
Dinoprostone vaginal insert alone	18 (13.9)	2 (12.5)				16 (13.7)	4 (13.8)			
Oral misoprostol alone	6 (4.6)	0				4 (3.4)	2 (6.9)			
Repeated cervical ripening methods	39 (30.0)	4 (25.0)				36 (30.8)	7 (24.1)			
CS	20 (15.4)	3 (18.8)	0.74			5 (4.3)	18 (62.1)	0.64		
Birth weight, g	3444 ± 436	3563 ± 377	0.29			3459 ± 406	3449 ± 554	0.91		

OR, odds ratio; CI, confidence interval; BMI, body mass index. Adjusted OR and 95% CI values were provided for the predictors for maternal and neonatal morbidity outcomes after cervical ripening that were considered as interest variables (*p*-value < 0.10 in univariate analysis and maternal age).

## Data Availability

The data presented in this study are available upon request from the corresponding author. The data are not publicly available due to institutional policy.

## References

[1] Collège national des gynécologues et obstétriciens français, Société francophone du diabète (2010). Gestational diabetes. J. Gynecol. Obstet. Biol. Reprod..

[2] Committee on Practice Bulletins—Obstetrics (2017). Practice bulletin no. 180: Gestational diabetes mellitus. Obstet. Gynecol..

[3] Sferruzzi-Perri A.N., Lopez-Tello J., Napso T., Yong H.E.J. (2020). Exploring the causes and consequences of maternal metabolic maladaptations during pregnancy: Lessons from animal models. Placenta.

[4] Di Cianni G., Miccoli R., Volpe L., Lencioni C., Del Prato S. (2003). Intermediate metabolism in normal pregnancy and in gestational diabetes. Diabetes Metab. Res. Rev..

[5] Barbour L.A., McCurdy C.E., Hernandez T.L., Kirwan J.P., Catalano P.M., Friedman J.E. (2007). Cellular mechanisms for insulin resistance in normal pregnancy and gestational diabetes. Diabetes Care.

[6] Buchanan T.A., Xiang A., Kjos S.L., Watanabe R. (2007). What Is Gestational Diabetes?. Diabetes Care.

[7] Al-Qahtani S., Heath A., Quenby S., Dawood F., Floyd R., Burdyga T., Wray S. (2012). Diabetes is associated with impairment of uterine contractility and high Caesarean section rate. Diabetologia.

[8] Catalano P.M., McIntyre H.D., Cruickshank J.K., McCance D.R., Dyer A.R., Metzger B.E., Lowe L.P., Trimble E.R., Coustan D.R., Hadden D.R. (2012). The Hyperglycemia and Adverse Pregnancy Outcome Study: Associations of GDM and obesity with pregnancy outcomes. Diabetes Care.

[9] Wong T., Ross G.P., Jalaludin B.B., Flack J.R. (2013). The clinical significance of overt diabetes in pregnancy. Diabet. Med..

[10] Shand A.W., Bell J.C., McElduff A., Morris J., Roberts C.L. (2008). Outcomes of pregnancies in women with pre-gestational diabetes mellitus and gestational diabetes mellitus; a population-based study in New South Wales, Australia, 1998–2002. Diabet. Med..

[11] Billionnet C., Mitanchez D., Weill A., Nizard J., Alla F., Hartemann A., Jacqueminet S. (2017). Gestational diabetes and adverse perinatal outcomes from 716,152 births in France in 2012. Diabetologia.

[12] Metzger B.E., Lowe L.P., Dyer A.R., Trimble E.R., Chaovarindr U., Coustan D.R., Hadden D.R., McCance D.R., Hod M., HAPO Study Cooperative Research Group (2008). Hyperglycemia and adverse pregnancy outcomes. N. Engl. J. Med..

[13] Swift E.M., Gunnarsdottir J., Zoega H., Bjarnadottir R.I., Steingrimsdottir T., Einarsdottir K. (2022). Trends in labor induction indications: A 20-year population-based study. Acta Obstet. Gynecol. Scand..

[14] Jozwiak M., Oude Rengerink K., Benthem M., van Beek E., Dijksterhuis M.G., de Graaf I.M., E van Huizen M., A Oudijk M., Papatsonis D.N., Perquin D.A. (2011). Foley catheter versus vaginal prostaglandin E2 gel for induction of labour at term (PROBAAT trial): An open-label, randomised controlled trial. Lancet.

[15] Diguisto C., Le Gouge A., Arthuis C., Winer N., Parant O., Poncelet C., Chauleur C., Hannigsberg J., Ducarme G., Gallot D. (2021). Cervical ripening in prolonged pregnancies by silicone double balloon catheter versus vaginal dinoprostone slow release system: The MAGPOP randomised controlled trial. PLoS Med..

[16] Ten Eikelder M.L., Neervoort F., Oude Rengerink K., van Baaren G.J., Jozwiak M., de Leeuw J.W., de Graaf I., van Pampus M.G., Franssen M., Oudijk M. (2013). Induction of labour with a Foley catheter or oral misoprostol at term: The PROBAAT-II study, a multicentre randomised controlled trial. BMC Pregnancy Childbirth.

[17] Jozwiak M., ten Eikelder M., Oude Rengerink K., de Groot C., Feitsma H., Spaanderman M., van Pampus M., de Leeuw J.W., Mol B.W., Bloemenkamp K. (2014). Foley catheter versus vaginal misoprostol: Randomized controlled trial (PROBAAT-M study) and systematic review and meta-analysis of literature. Am. J. Perinatol..

[18] ten Eikelder M.L.G., Mast K., van der Velden A., Bloemenkamp K.W.M., Mol B.W. (2016). Induction of Labor Using a Foley Catheter or Misoprostol: A Systematic Review and Meta-analysis. Obstet. Gynecol. Surv..

[19] Mozurkewich E.L., Chilimigras J.L., Berman D.R., Perni U.C., Romero V.C., King V.J., Keeton K.L. (2011). Methods of induction of labour: A systematic review. BMC Pregnancy Childbirth.

[20] de Vaan M.D., Ten Eikelder M.L., Jozwiak M., Palmer K.R., Davies-Tuck M., Bloemenkamp K.W., Mol B.W.J., Boulvain M. (2023). Mechanical methods for induction of labour. Cochrane Database Syst. Rev..

[21] Vince K., Perković P., Matijević R. (2020). What is known and what remains unresolved regarding gestational diabetes mellitus (GDM). J. Perinat. Med..

[22] Timofeev J., Huang C.-C., Singh J., Driggers R.W., Landy H.J. (2012). Spontaneous labor curves in women with pregnancies complicated by diabetes. J. Matern. Fetal. Neonatal. Med..

[23] Jiang T., Zhao L., Lin Y., Zhou D., Wang L., Sun G., Xiao M. (2019). Effects of gestational diabetes mellitus on time to delivery and pregnancy outcomes in full-term pregnancies with dinoprostone labor induction. Clin. Exp. Hypertens..

[24] Rayburn W.F., Sokkary N., Clokey D.E., Moore L.E., Curet L.B. (2005). Consequences of routine delivery at 38 weeks for A-2 gestational diabetes. J. Matern. Fetal. Neonatal. Med..

[25] Nevander S., Carlhäll S., Källén K., Lilliecreutz C., Blomberg M. (2023). Gestational diabetes mellitus and time in active labor: A population-based cohort study. Acta Obstet. Gynecol. Scand..

[26] Vitner D., Hiersch L., Ashwal E., Shmueli A., Yogev Y., Aviram A. (2019). Induction of labor versus expectant management for gestational diabetes mellitus at term. Arch. Gynecol. Obstet..

[27] Melamed N., Ray J.G., Geary M., Bedard D., Yang C., Sprague A., Murray-Davis B., Barrett J., Berger H. (2016). Induction of labor before 40 weeks is associated with lower rate of cesarean delivery in women with gestational diabetes mellitus. Am. J. Obstet. Gynecol..

[28] Vilchez G.A., Dai J., Hoyos L.R., Gill N., Bahado-Singh R., Sokol R.J. (2015). Labor and neonatal outcomes after term induction of labor in gestational diabetes. J. Perinatol..

[29] Worda K., Bancher-Todesca D., Husslein P., Worda C., Leipold H. (2017). Randomized controlled trial of induction at 38 weeks versus 40 weeks gestation on maternal and infant outcomes in women with insulin-controlled gestational diabetes. Wien. Klin. Wochenschr..

[30] Ducarme G., Martin S., Chesnoy V., Planche L., Berte M.P., Netier-Herault E. (2022). Prospective observational study investigating the effectiveness, safety, women’s experiences and quality of life at 3 months regarding cervical ripening methods for induction of labor at term—The MATUCOL study protocol. PLoS ONE.

[31] Ducarme G., Desroys Du Roure F., Le Thuaut A., Grange J., Dimet J., Crepin-Delcourt I. (2018). Efficacy of maternal and biological parameters at the time of diagnosis of gestational diabetes mellitus in predicting neonatal morbidity. Eur. J. Obstet. Gynecol. Reprod. Biol..

[32] Ducarme G., Desroys du Roure F., Grange J., Vital M., Le Thuaut A., Crespin-Delcourt I. (2019). Predictive factors of subsequent insulin requirement for glycemic control during pregnancy at diagnosis of gestational diabetes mellitus. Int. J. Gynaecol. Obstet..

[33] Ego A. (2013). Definitions: Small for gestational age and intrauterine growth retardation. J. Gynecol. Obstet. Biol. Reprod..

[34] Committee on Practice Bulletins—Obstetrics (2017). Practice Bulletin No 178, Shoulder Dystocia. Obstet. Gynecol..

[35] Palatnik A., Grobman W.A., Hellendag M.G., Janetos T.M., Gossett D.R., Miller E.S. (2016). Predictors of shoulder dystocia at the time of operative vaginal delivery. Am. J. Obstet. Gynecol..

[36] Lyndrup J., Legarth J., Weber T., Nickelsen C., Guldbæk E. (1992). Predictive value of pelvic scores for induction of labor by local PGE2. Eur. J. Obstet. Gynecol. Reprod. Biol..

[37] Ivars J., Garabedian C., Devos P., Therby D., Carlier S., Deruelle P., Subtil D. (2016). Simplified Bishop score including parity predicts successful induction of labor. Eur. J. Obstet. Gynecol. Reprod. Biol..

[38] Duffy J.Y., Chau C., Raymond K., Rugarn O., Wing D.A. (2023). The Influence of Diabetes on Labor Induction with Dinoprostone Vaginal Inserts. Am. J. Perinatol..

